# Inbreeding reveals mode of past selection on male reproductive characters in *Drosophila melanogaster*

**DOI:** 10.1002/ece3.625

**Published:** 2013-06-03

**Authors:** Outi Ala-Honkola, David J Hosken, Mollie K Manier, Stefan Lüpold, Elizabeth M Droge-Young, Kirstin S Berben, William F Collins, John M Belote, Scott Pitnick

**Affiliations:** 1 Department of Biology, Syracuse UniversitySyracuse, New York; 2 Department of Biological and Environmental Science, University of JyväskyläPO Box 35, 40014, Finland; 3 Centre for Ecology and Conservation, College of Life and Environmental Sciences, University of ExeterExeter, Devon, United Kingdom

**Keywords:** Attractiveness, *Drosophila melanogaster*, inbreeding depression, past selection, sperm competition, sperm length

## Abstract

Directional dominance is a prerequisite of inbreeding depression. Directionality arises when selection drives alleles that increase fitness to fixation and eliminates dominant deleterious alleles, while deleterious recessives are hidden from it and maintained at low frequencies. Traits under directional selection (i.e., fitness traits) are expected to show directional dominance and therefore an increased susceptibility to inbreeding depression. In contrast, traits under stabilizing selection or weakly linked to fitness are predicted to exhibit little-to-no inbreeding depression. Here, we quantify the extent of inbreeding depression in a range of male reproductive characters and then infer the mode of past selection on them. The use of transgenic populations of *Drosophila melanogaster* with red or green fluorescent-tagged sperm heads permitted in vivo discrimination of sperm from competing males and quantification of characteristics of ejaculate composition, performance, and fate. We found that male attractiveness (mating latency) and competitive fertilization success (P_2_) both show some inbreeding depression, suggesting they may have been under directional selection, whereas sperm length showed no inbreeding depression suggesting a history of stabilizing selection. However, despite having measured several sperm quality and quantity traits, our data did not allow us to discern the mechanism underlying the lowered competitive fertilization success of inbred (*f* = 0.50) males.

## Introduction

Mating between close relatives often leads to a decrease in fitness known as inbreeding depression (Lynch and Walsh 1998), which can be strong enough to drive small populations to extinction (Saccheri et al. 1998; O'Grady et al. 2006). Understanding the effects of inbreeding on reproductive success is becoming increasingly important, as many animal populations become smaller and more fragmented, thus increasing the likelihood of mating between close relatives (Frankham et al. 2002). Inbreeding depression can be caused by either the loss of high-fitness heterozygotes (the overdominance hypothesis) or by increased expression of deleterious recessives (the partial dominance hypothesis) (Lynch and Walsh 1998). Inbreeding increases homozygosity, and according to the overdominance hypothesis, the decrease in the frequency of high-fitness heterozygotes leads to a decline in fitness. The partial dominance hypothesis (that currently has the greatest support; Charlesworth and Willis 2009) proposes that increasing homozygosity unmasks deleterious recessive alleles leading to a fitness decline.

Directional dominance is required for inbreeding depression (Falconer and Mackay 1996; Roff 1997; Lynch and Walsh 1998). Directionality arises due to natural or sexual selection driving alleles that increase fitness to fixation and eliminating dominant deleterious alleles, whereas deleterious recessive alleles are hidden from selection and hence maintained at low frequencies (Falconer and Mackay 1996; Roff 1997; Lynch and Walsh 1998). These deleterious recessives are then expressed in inbred individuals that are more homozygous than outbred individuals (Falconer and Mackay 1996; Roff 1997; Lynch and Walsh 1998). Thus, traits closely linked to fitness are predicted to show strong inbreeding depression (Falconer and Mackay 1996; Roff 1997; Lynch and Walsh 1998) and several studies support this prediction (Falconer and Mackay 1996; Roff 1998; DeRose and Roff 1999; Wright et al. 2008; but see Ellmer and Andersson 2004). In contrast, directional dominance should be low for traits weakly associated with fitness or those under stabilizing selection because mutations moving trait values up or down will be selectively equivalent and hence such traits are predicted to exhibit little-to-no inbreeding depression (Lynch and Walsh 1998). Therefore, inbreeding depression in a trait is a signature of directional selection in the past, whereas a lack of inbreeding depression suggests either stabilizing or weak selection in the past (Falconer and Mackay 1996; Roff 1997; Lynch and Walsh 1998; Ketola et al. in press).Distinguishing stabilizing selection from weak selection based on the lack of inbreeding depression is not possible without further knowledge on selection acting on the trait in question. Also, it is important to bear in mind that recessive alleles in very important fitness traits may have been purged under small population size by strong directional selection, which can substantially lower inbreeding depression (Falconer and Mackay 1996; Ketola et al. in press). In addition, the degree of inbreeding depression is nonlinearly dependent on the frequency of the recessive alleles in the population: the strongest inbreeding depression occurs when the frequency of recessive alleles is intermediate (Falconer and Mackay 1996). Thus, the differences in the magnitude of the inbreeding depression can also depend on the numbers of loci coding for traits. As the number of loci involved in trait expression increases, selection per locus weakens, and this weakening maintains recessive alleles in higher frequencies and results in higher inbreeding depression in the trait (Falconer and Mackay 1996). Inbreeding depression is further affected by the genomic mutation rate U, with higher U causing stronger inbreeding depression (Roff 1997).

For traits that have been under directional selection, the partial dominance hypothesis of inbreeding depression predicts that inbreeding moves trait values away from high fitness because deleterious recessive alleles will always change trait values in the direction opposing the long-term past selection (Falconer and Mackay 1996; Roff 1997; Lynch and Walsh 1998; Ketola et al. in press). Hence the direction of inbreeding depression can be used to identify trait values associated with high fitness, although this approach has not been widely applied (but see Mackay 1985; Mallet and Chippindale 2011; Ketola and Kotiaho 2012). Here, we aim to better understand the evolution of sperm length using the inbreeding method. Even though comparative investigations in a wide range of taxa have found relationships between sperm length and the level of sperm competition (reviewed by Snook 2005; Pitnick et al. 2009a; Pizzari and Parker 2009), selection acting on sperm length is not well understood in most taxa, despite sperm competition being widespread and credited with driving the rapid diversification of ejaculate traits (Parker 1970; Ravi Ram and Wolfner 2007; Pitnick et al. 2009a,b; Pizzari and Parker 2009). Thorough investigation of sperm length variation in *Drosophila*, however, using comparative, experimental, genetic, and functional approaches has demonstrated rapid diversification of sperm length (Pitnick et al. 1999, 2003; Manier et al. 2013). Longer sperm have a selective advantage because they are better at displacing, and resisting displacement, by competitor sperm from female sperm storage organs (Miller and Pitnick 2002; Pattarini et al. 2006; Lüpold et al. 2012; M. K. Manier, J. M. Belote, S. Lüpold, O. Ala-Honkola, K. S. Berben, W. T. Starmer and S. Pitnick, unpubl. data), and sperm length has a close association with the intensity of sexual selection (Bjork and Pitnick 2006). If there is strong directional selection for longer sperm, as experimental studies suggest, we would expect inbreeding to decrease sperm length. On the other hand, substantive energetic and life-history costs of manufacturing relatively long sperm have been demonstrated (Pitnick et al. 1995; Pitnick 1996; Immler et al. 2011), and thus net selection on sperm length may be stabilizing. Indeed, a hemiclonal analysis of *D. melanogaster* found high heritability but low evolvability (the coefficient of additive genetic variation; Houle 1992) of sperm length and concluded this pattern was consistent with stabilizing selection (Morrow et al. 2008). In this case, theory predicts that directional dominance for sperm length will be low and that there will be no inbreeding depression in sperm length (Roff 1997; Lynch and Walsh 1998).

Several recent studies have shown that inbreeding typically decreases sperm competitiveness (Hughes 1997; Konior et al. 2005; Zajitschek et al. 2009; Michalczyk et al. 2010; Simmons 2011). However, the mechanisms leading to the lower fertilization success of inbred males remain unresolved. Correlational studies documented that sperm number and quality (e.g., sperm motility or proportion of morphologically normal sperm in an ejaculate) are often lower in inbred populations (Wildt et al. 1982; Roldan et al. 1998; Gomendio et al. 2000; Margulis and Walsh 2002; van Eldik et al. 2006; Gage et al. 2006; Fitzpatrick and Evans 2009; Weeks et al. 2009). In addition, an experimental investigation of the guppy, *Poecilia reticulata*, showed that inbreeding decreases sperm numbers (Zajitschek and Brooks 2010), but such an effect was not found in three-spined sticklebacks, *Gasterosteus aculeatus* (Mehlis et al. 2012). Despite the obvious connection between sperm traits and sperm competition success, experimental studies that simultaneously measure the effects of inbreeding on competitive fertilization success and characteristics of the sperm themselves are currently lacking.

In addition to sperm, other male reproductive characters are closely linked to fitness and therefore should also be sensitive to inbreeding depression. Male mating success has been shown to be a major fitness component in *D. melanogaster* (Prout 1971a,b; Bundgaard and Christiansen 1972) and, as predicted, it decreases with inbreeding (Brittnacher 1981; Sharp 1984; Partridge et al. 1985; Miller et al. 1993; Hughes 1995; Enders and Nunney 2010). Reduced male mating success due to inbreeding has also been reported in the housefly *Musca domestica* (Meffert and Bryant 1991), the butterfly *Bicyclus anynana* (Joron and Brakefield 2003), two species of poeciliid fish (van Oosterhout et al. 2003; Mariette et al. 2006; Ala-Honkola et al. 2009) and in the decorated cricket *Gryllodes sigillatus* (Ketola and Kotiaho 2010). In the fly *D. simulans*, inbreeding reduced male attractiveness (Okada et al. 2011), as measured by copulation latency, a standard measure of attractiveness (attractive males mate faster: Ritchie et al. 1999; Barth et al. 1997; Taylor et al. 2008). Inbred males have also been shown to be less attractive in the house mouse *Mus musculus* (Ilmonen et al. 2009), the zebra finch *Taeniopygia guttata* (Bolund et al. 2010), the guppy *P. reticulata* (Zajitschek and Brooks 2010), and the meal worm beetle *Tenebrio molitor* (Pölkki et al. 2012).

Here, we report on an investigation of the influence of inbreeding on male attractiveness (mating latency) and competitive fertilization success in *D. melanogaster*. We also assess the impact of inbreeding on several characters that affect sperm competition success, such as sperm length, ejaculate size, in vivo sperm swimming speed, as well as sperm viability and sperm storage in the female reproductive tract, with the goal of inferring the mode of past selection on all these traits. These characters are all key determinants of male reproductive success and hence should be closely linked to fitness. Indeed, previous work has shown that male reproductive success is the most important meta-trait determining male fitness (Prout 1971a,b; Bundgaard and Christiansen 1972), and sperm competitiveness and sperm length have been shown to directly determine male fitness in *D. melanogaster* (Miller and Pitnick 2002; Pattarini et al. 2006; Fricke et al. 2010; Lüpold et al. 2012). Ejaculate size has been shown to positively correlate with the amount of previous male's sperm displaced in the same population as in this study (Manier et al. 2010) suggesting selection for larger ejaculate size. Also, relatively slow and/or long sperm have been shown to be better at displacing resident sperm from storage (Lüpold et al. 2012) suggesting selection for slower sperm. By assessing inbreeding depression or lack thereof in these traits, we can infer the history of past selection acting on them: inbreeding depression implies a history of directional selection (to generate the directional dominance needed to cause inbreeding depression), whereas a lack of inbreeding depression implies stabilizing or weak selection (as mutations up or down are selectively equivalent).

**Figure 1:**
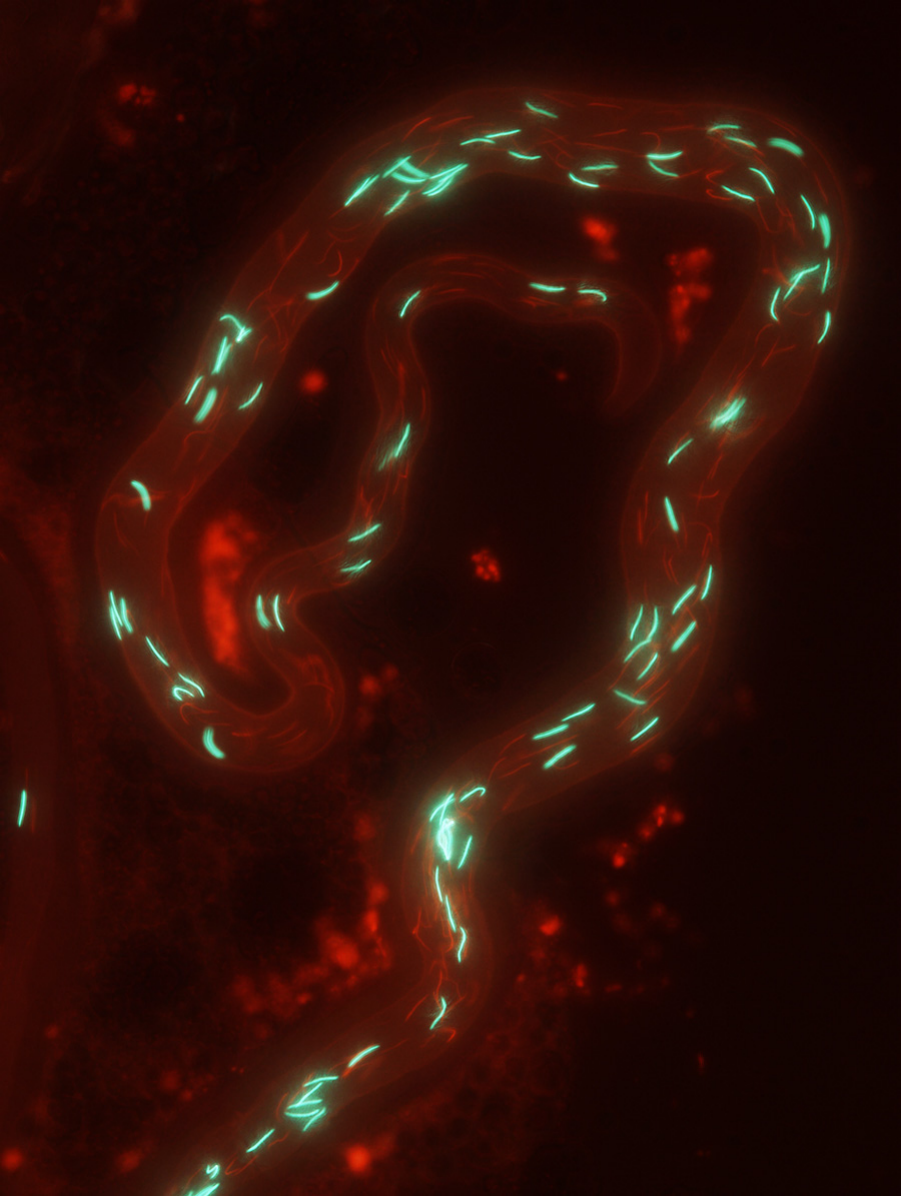
*Drosophila melanogaster* lines with red or green fluorescently tagged sperm heads allowed us to distinguish between the ejaculates of two males in competition within the female reproductive tract (here inside female seminal receptacle).

The use of transgenic flies with either red or green fluorescently tagged sperm heads (Manier et al. 2010) allowed us to distinguish between the ejaculates of two males in competition within the female reproductive tract and to quantify aspects of ejaculate quality and fate (Fig. 1, Manier et al. 2010; Lüpold et al. 2011, 2012). As the severity of inbreeding depression in a population depends (among other things) on historical population size and ancestral inbreeding, its level is difficult to predict a priori (Falconer and Mackay 1996; Frankham et al. 2002). Both Zajitschek et al. (2009) and Robinson et al. (2009) did not find inbreeding depression in sperm competition success at low levels on inbreeding (*f* = 0.25 or less), so we used two levels of inbreeding in this study: a theoretical *f* = 0.25 (one generation of full-sibling breeding) represents a realistic level of inbreeding in nature (Keller and Waller 2002) and *f* = 0.5 (three generations of full-sibling breeding) represents severe inbreeding.

## Materials and Methods

### Experimental populations

**Figure 2:**
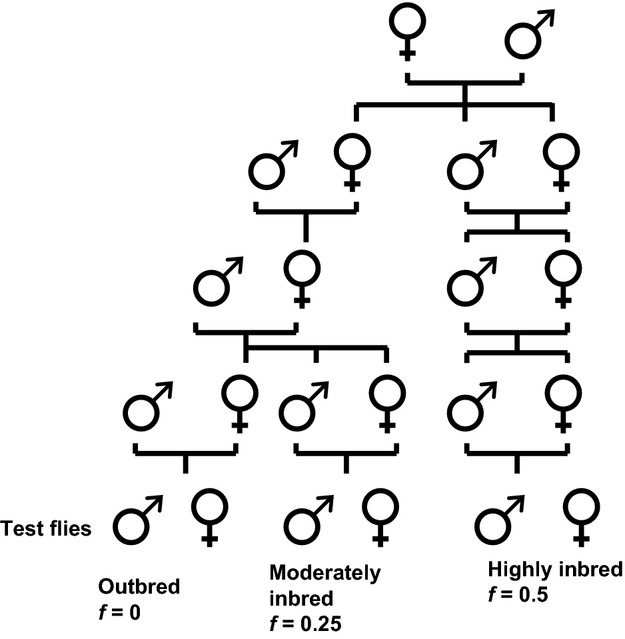
Breeding design to create flies with different inbreeding coefficients.

The experimental flies originated from a line genetically engineered to produce sperm with heads tagged by a red fluorescent protein (RFP; DsRed-Monomer) that were backcrossed for six generations to the LH_M_ wild-type strain (for details on the fly strains and the genetic transformation methods, see Manier et al. 2010). We generated lines of flies that differed in their inbreeding coefficient (Fig. 2) by mating full siblings for either three (highly inbred lines, theoretical *f* = 0.5), one (moderately inbred lines, theoretical *f* = 0.25) or zero generations (outbred control lines), following Zajitschek et al. (2009). All lines (with one back-up for each line) originated from 60 full-sibling families (F_0_), which were founded by placing pairs of randomly selected virgin females and males from the RFP-line into plastic 8-dram vials containing cornmeal-molasses-agar-yeast medium (5.4% cornmeal, 7% molasses, 0.5% agar, 2% yeast, 1.2% ethanol, 0.4% propionic acid, 0.06% methylparaben added to water) and a few grains of live yeast. F_1_ progeny from these families were randomly selected to the three inbreeding treatments (Fig. 2). To generate the outbred control lines, a virgin female from a given line was mated to a male from a randomly selected outbred line. To generate *f* = 0.25 flies, a female from a given line was mated to a male from a randomly selected outbred line in F_1_ and F_2_ and with a sibling in F_3_. For *f* = 0.5 flies, virgin females were mated to a full-sibling male in each generation. In F_4_, we had 56 lines in each treatment. During culturing, each pair was transferred to a new vial three times a week to avoid larval crowding. Virgin females and males for the experiments were collected under CO_2_ anesthetization.

To test the effects of inbreeding on male traits, males were mated to LH_M_ (Chippindale et al. 2001) wild-type females. LH_M_ is maintained in population cages of approximately 1000 individuals with overlapping generations at 24°C and 12L:12D. As standard competitors, we used males from a population with GFP-tagged sperm heads and GFP-tagged ubiquitin, which permits unambiguous paternity assignment by viewing adult offspring with a fluorescent stereomicroscope. These flies were bottle-reared and collected as virgins under CO_2_ anesthetization.

All experimental flies were 3–5 days old at their first experimental mating. In order to remove variation in P_2_ and sperm traits attributable to the males’ mating history (Bjork et al. 2007), all test and competitor males were each mated to a nonexperimental virgin female one day before their first experimental mating. Thorax length of all males and females were measured to the nearest 0.01 mm using the reticule of a stereomicroscope at 80× magnification.

### Single-mating productivity

To estimate the effect of inbreeding on male attractiveness, sperm viability within female reproductive tract and the viability of offspring (e.g., due to DNA fragmentation; Ruiz-Lopez et al. 2010), we mated one male from each experimental line (*N* = 56 in each treatment) to a virgin LH_M_ wild-type female. Pairs were aspirated into fresh plastic vials with 10 mL of medium and observed continuously. We recorded time from the introduction of the male to the start of copulation (i.e., mating latency, a proxy for male attractiveness) and copulation duration for all matings. Females were transferred to fresh vials with oviposition medium every day for 10 days. Egg-to-adult viability was estimated from eggs laid on days 1, 3, and 5 by counting the numbers of eggs laid and the number of enclosed adults per vial.

### Ejaculate size, sperm storage, and P_2_

An experiment was conducted to quantify (1) the number of sperm transferred, (2) the number of sperm stored by females, and (3) the proportion of offspring sired by the second male to mate (P_2_). On day 0, each virgin female was mated with a standard competitor male; on day 2, females were given the first opportunity to remate (4-h time window); any refractory females were provided additional opportunities on days 3–5 until remating occurred. We counted all the progeny (enclosed adults) produced prior to remating (results reported for P_2_ only) to ensure the first mating was successful and to estimate sperm usage of first-male sperm prior to remating. All matings were performed in one large replicate, having one inbred male per line in each experiment (i.e., sperm ejaculated, sperm stored, and P_2_). As above, pairs were aspirated into fresh food vials, observed continuously, and the copulation duration and remating day were recorded for each mating.

The number of sperm ejaculated was determined using females that were flash-frozen in liquid nitrogen 60 min after the second copulation started (copulations last approximately 20 min) and the amount of sperm stored by females was determined using females flash-frozen 5 h after the second copulation started (i.e., after females have ejected a mass containing excess second-male sperm and displaced first-male sperm; see Manier et al. 2010). The proportion of first-male sperm that the second male displaced was counted as the number of first-male sperm in the bursa (i.e., those that will be ejected later) divided by the total number of first-male sperm in the reproductive tract at 60 min samples (i.e., before the ejection of excess sperm, Manier et al. 2010). We also counted first-male sperm in the female reproductive tract in specimens of the 5-h and 6-day treatments. P_2_ was calculated from offspring produced over 6 days after remating, with females transferred to fresh vials after 1 and 3 days and frozen after 6 days until quantification of the sperm remaining in the reproductive tracts after 6 days of egg-laying. All females were stored at −20°C until dissection.

For sperm counts, we dissected the female reproductive tract into a drop of phosphate-buffered saline (PBS) on a microscope slide and unfolded the seminal receptacle (SR) before covering the specimen with a coverslip and sealing it with paper cement. Under a fluorescent microscope at a magnification of 400×, the number of sperm in the bursa, SR, and the two spermathecae with ducts were counted. All sperm counts were done blind with respect to the treatment. The number of sperm ejaculated was the total number of second-male sperm (red) in the female reproductive tract. For the number of sperm stored, we report both the number of second-male sperm in the SR and the SR and the paired spermathecae combined. We dissected 25–35 females per treatment in each experiment.

### Sperm swimming speed

In vivo sperm swimming speed was recorded in reproductive tracts of once-mated females (*N* = 20 per inbreeding treatment) dissected 120 min after the start of the copulation. Females were anesthetized with CO_2_ and their reproductive tracts were removed as described above and mounted under a coverslip in 20 μL (to standardize tract compression) of Grace's Supplemented Insect Medium (Invitrogen, Paisley, U.K.) at room temperature. Ten second-long movies (74 frames; AVI) were recorded within 3–7 min of anesthetization using an Olympus DP71 digital camera and DPController Software version 3.3.1.292 (Olympus America Inc., Center Valley, PA).

Each movie was imported into NIH ImageJ (v. 1.42q, National Institutes of Health, Bethesda, MD, http://rsb.info.nih.gov/ij/) as a monochrome stack, which was then inverted from a dark background to a light background. We measured slice-by-slice instantaneous linear velocities (μm/sec) for 10 sperms per male using the Manual Tracking plugin for ImageJ (available at http://rsbweb.nih.gov/ij/plugins/index.html). Sperm counts of the entire seminal receptacle were also obtained for each female as described above to statistically account for density effects on swimming speed (Manier et al. 2010; Lüpold et al. 2012). Average instantaneous velocities were calculated per tracked sperm.

### Sperm length

Sperm length was measured from 22 lines per treatment (one male per line). Following ether anesthetization of a male, we dissected one seminal vesicle into PBS on a subbed microscope slide and then ruptured it with a fine probe. Sperm were dispersed in the droplet before drying the slide at 60°C, fixing in methanol:acetic acid (3:1), rinsing in PBS and mounting under a coverslip in glycerol and PBS (80/20 v/v). We measured dark-field images of six sperm per male at 200× magnification by tracing with the segmented line tool of ImageJ v. 1.44j (National Institutes of Health, U.S.A.). All measurements were done blind with respect to the treatment. The longest and the shortest sperm per male were left out of the analysis in order to avoid including broken sperm tails in the analysis (i.e., four sperms per male were used to calculate means). Males were 2–3 weeks old at the time of dissection.

### Data analyses

To compare inbreeding depression between different traits, we calculated the standardized coefficient of inbreeding, δ, by dividing the difference in mean trait values between outbred and inbred individuals by the mean trait value of outbred individuals (Lande and Schemske 1985). We used R 2.12.0 for statistical analysis (R Development Core Team 2010) except that Tukey post hoc tests were conducted with function glht (library multcomp) in R version 2.15.2.

### Time series analyses of progeny production, offspring viability, and P_2_ data

Single-mating productivity, offspring viability, and P_2_ data consist of repeated measures of the same individuals at regularly spaced time-points. These data were analyzed with generalized least squares (GLS) models (function gls in the library nlme in R). Only complete time series were included in the analyses. Males were excluded from the P_2_ analyses if the female did not produce any offspring after the first mating or if the female had a P_2_ value of “0,” because these occurrences are symptomatic of an unsuccessful copulation.

Male and female thorax length, and treatment (inbreeding level) × time interaction were entered as fixed factors into the full models. We tested different variance covariance structures between observations from the same individuals (compound symmetry, first-order autoregressive and first-order autoregressive with heterogeneous variances) and chose the one that best fit the data based on AIC values (see Statistical Consulting Group; Diggle et al. 2009). P_2_ values were arcsine square root-transformed as they are proportions. Viabilities, however, were not transformed as we had several values over 1 as viability where eggs were missed while counting.

The optimal fixed structure of the models was determined by comparing nested models using likelihood ratio (L-ratio) tests (maximum likelihood, ML) and the final model was refitted with restricted maximum likelihood (REML) estimation as suggested by Zuur et al. (2009). We performed model validations by examining the homogeneity and independence of errors. See Tables S2–S4 for full models of time series analyses.

### Mating latency, sperm numbers, and progeny production before and after remating

We used general linear (function lm in R) or GLS models to analyze the effect of inbreeding on male mating latency, copulation durations with virgin females, sperm numbers, and progeny production before and after remating. In several cases, variance increased with inbreeding (see SDs in Table 1) and using treatment as a variance covariate (function varIdent in R) significantly improved those models based on likelihood ratio tests (Zuur et al. 2009). If male or female thorax lengths were not correlated with the dependent variable, they were removed from the models. Thus, we typically only fitted treatment as a factor into our models. Mating latency and the number of first-male sperm in storage 6 days after remating were log_10_-transformed to avoid heteroscedasticity in residuals and the proportion of first-male sperm displaced was arcsine square root-transformed.

**Table 1:** Effects of inbreeding on measured male traits

Trait	Mean (SD), *N*		

	Outbred (*f* = 0)	Moderately inbred (*f* =0.25)	Highly inbred (*f* = 0.5)
Mating latency (min)	24.5 (35.7), 52	40.4 (53.4), 53	45.8 (56.8), 49
Remating day	3.25 (0.54), 148	3.39 (0.65), 147	3.44 (0.67), 153
Progeny production before remating	73.6 (36), 48	72.0 (33), 46	92.1 (45), 48
Progeny production after remating	217 (45.1), 48	212 (42.9), 46	217 (50.0), 48
Copulation duration with virgin (min)	21.1 (5.7), 52	19.9 (4.4), 51	20.2 (5.3), 49
Copulation duration with nonvirgin (min)	25.4 (6.1), 147	23.8 (5.8), 146	24.8 (5.7), 151
Sperm ejaculated by the second male	1160 (295), 32	1170 (322), 33	1130 (258), 34
Proportion of first male's sperm displaced	0.23 (0.32), 28	0.36 (0.33), 27	0.30 (0.27), 33
Second male's sperm stored in SR	279 (46), 24	271 (67), 24	291 (92), 24
Second male's sperm stored in SR and SPTH	360 (78), 24	351 (91), 24	380 (130), 24
First male's sperm in reproductive tract (5 h ASM)	14.7 (19.9), 24	27.0 (32.6), 24	30.0 (34.7), 24
Second male's sperm in female reproductive tract after 6 days ASM	124 (93.3), 25	166 (107), 23	132 (101), 25
First male's sperm in female reproductive tract 6 days ASM	12.4 (27.7), 25	23.6 (43.2), 23	9.48 (20.9), 25
Sperm length (mm)	1.78 (0.051), 22	1.76 (0.059), 22	1.76 (0.074), 21
Sperm swimming speed (μm/sec)	28.7 (13.7), 20	28.4 (13.7), 20	30.0 (19.0), 20

Data for mating latency and copulation duration with virgin are from the single-mating productivity experiment. SR, seminal receptacle; SPTH, spermathecae; ASM, after the start of the second mating.

### Sperm length, sperm swimming speed, and copulation durations during remating

Sperm length, sperm swimming speed, and copulation durations during rematings and remating day were analyzed with general linear mixed models (function lme in library nlme in R) because we had several measurements per male (sperm length, sperm swimming speed; male as a random factor) or per line (remating day, copulation duration; line as a random factor). The random factor was significant (assessed using likelihood ratio tests, Zuur et al. 2009) only in the analyses of sperm length and sperm swimming speed but to be conservative, we kept it also in all analyses to avoid pseudo-replication.

In all analysis, male and female thorax lengths and treatment (and in the analyses of sperm swimming speed, also the number of sperm in SR) were entered as fixed factors into the full model. Sperm swimming speed was log_10_-transformed to avoid heteroscedasticity in residuals. The optimal fixed structure of the models was determined as above.

### Retrospective power analysis

In order to estimate the power of our sperm trait analyses, we followed Thomas's (1997) suggestions and estimated the power based on prespecified effect size for sperm characters. We suggest that 10% change in trait values between outbred and inbred individuals is biologically meaningful as it equals 10% inbreeding depression and can thus be considered severe inbreeding depression. For sperm numbers, power calculations were straightforward by being simple analyses of variance (ANOVAs), and power could be estimated following Zar (1999, p. 192). For sperm swimming speed and sperm length, as well as offspring production after single mating, we took the confidence interval approach (Thomas 1997) because power calculations for mixed models and repeated measures designs are very complicated. In brief, we checked whether a 10% change from the outbred treatment's mean value would fall outside the 95% confidence interval of outbred treatment's means.

## Results

### Effects of inbreeding on mating behavior

There was a significant effect of inbreeding level on male attractiveness (mating latency; *F*_2,151_ = 5.43, *P* = 0.005), with inbred males being less attractive (i.e., taking longer to mate) than outbred males (see means and SDs in Table 1; outbred vs. moderately inbred Tukey *P* = 0.048; outbred vs. highly inbred Tukey *P* = 0.005, moderately inbred vs. highly inbred Tukey *P* = 0.69). The standardized coefficient of inbreeding (δ) was 65% for moderately inbred males and 87% for highly inbred males. There was also a significant effect of inbreeding status on remating speed (L-ratio for treatment = 7.85, df = 2, *P* = 0.020), with highly inbred males being slower to remate than outbred males (δ = 6%), but the difference between moderately inbred males and outbred males was not significant (see means and SDs in Table 1; outbred vs. moderately inbred Tukey *P* = 0.11; outbred vs. highly inbred Tukey *P* = 0.019, moderately inbred vs. highly inbred Tukey *P* = 0.77). The effect of slower remating speed of highly inbred males was also seen in progeny production before remating; females produced more offspring before remating when the second male was highly inbred, largely because the time frame for offspring production was longer (see means and SDs for progeny production before remating in Table 1; *F*(treatment)_2,139_ = 3.97, *P* = 0.021; outbred vs. moderately inbred Tukey *P* = 0.98; outbred vs. highly inbred Tukey *P* = 0.053, moderately inbred vs. highly inbred Tukey *P* = 0.034). Progeny production after remating did not differ among inbreeding levels (Table 1; *F*(treatment)_2,138_ = 0.14, *P* = 0.87). Copulation duration did not differ among inbreeding levels when males mated with virgins (Table 1; *F*(treatment)_2,149_ = 0.78, *P* = 0.46) or with once-mated females (L-ratio for treatment = 4.90, df = 2, *P* = 0.08, see also Table 1).

### Single-mating productivity and egg-to-adult viability

There was no difference between inbred and control-line males in the fertility of their mates following a single insemination (L-ratio for treatment × time interaction = 24.70, df = 24, *P* = 0.13 and L-ratio for treatment = 3.38, df = 2, *P* = 0.18, see also Table S1 and Fig. S1), which suggests that inbred males’ sperm survive equally well inside the female reproductive tract and fertilize eggs as efficiently as the sperm of outbred males (we would have been able to detect a 10% change in offspring production). Also, the offspring of inbred males did not suffer from decreased viability (L-ratio for treatment × time interaction = 9.02, df = 4, *P* = 0.06 and L-ratio for treatment = 1.92, df = 2, *P* = 0.38, Intercept [0.94, SE 0.01, residual df = 371] was the only term to remain in the GLS AR1 model, see also Fig. S2).

### Effects of male inbreeding on number of sperm ejaculated, sperm storage, and P_2_

**Figure 3:**
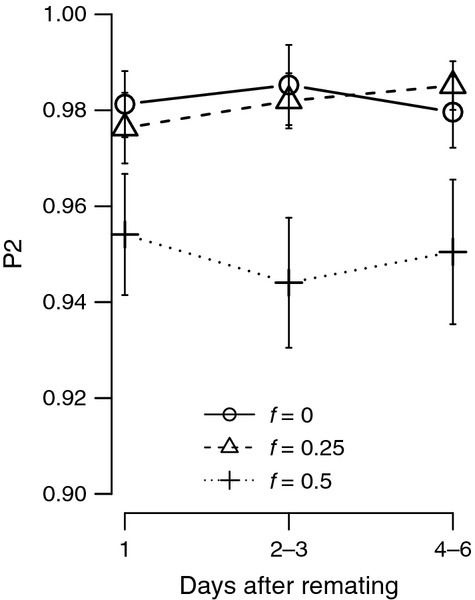
Proportion of offspring sired by the second male to mate (P_2_; mean ± SE) when second males were inbred to different degrees and first males were outbred competitor males (*N* = 48 in *f* = 0 and *f* = 0.5, *N* = 46 in *f* = 0.25).

Highly inbred males had lower competitive fertilization success (P_2_) compared to outbred males (δ = 3%), but there was no difference between outbred males and moderately inbred males (Fig. 3, L-ratio for treatment = 6.08, df = 2, *P* = 0.047; outbred vs. moderately inbred Tukey *P* = 0.98; outbred vs. highly inbred Tukey *P* = 0.046, moderately inbred vs. highly inbred Tukey *P* = 0.070; see also Table 2 for the final model). However, we saw no difference in any of the sperm traits measured among inbred and outbred males that could explain the lower fertilization success of highly inbred males (see means and SDs in Table 1). Specifically, the number of second-male (i.e., focal-male) sperm ejaculated (*F*_2,96_ = 0.23, *P* = 0.79), the proportion of first-male sperm displaced (*F*_2,85_ = 1.66, *P* = 0.20), the number of second-male sperm females stored in the SR (*F*_2,69_ = 0.34, *P* = 0.71) or in the SR and spermathecae combined (*F*_2,69_ = 0.41, *P* = 0.66) did not differ among inbreeding levels. The number of first-male sperm that was left in female reproductive tract 5 h after the second mating, did not differ among inbreeding levels (*F*_2,69_ = 2.39, *P* = 0.10). Similarly, there was no difference in long-term sperm storage among inbreeding treatments: the number of second-male sperm (*F*_2,70_ = 1.20, *P* = 0.31) and the number of first-male sperm (*F*_2,68_ = 0.13, *P* = 0.87) in the female reproductive tract 6 days after remating did not differ among inbreeding levels. However, the power to detect differences in sperm counts was very low due to large variation within treatments. For the number of sperm ejaculated, we would have only been able to detect a 20% change at power >0.80 and for the number of second-male sperm stored (both in the SR and in the spermathecae and SR combined) we would have only been able to detect a 30% change at power level >0.80. For the rest of the sperm counts, power was even lower.

**Table 2:** Final least squares model of sperm competition success (P_2_) of inbred males (first-order autoregressive variance covariance structure [AR1] and “treatment” as a variance covariate)

Effect	Parameter estimate	SE of the estimate	*t*	*P*
Intercept (arc sin sqrt transformed)	1.50	0.015	98	<0.000
Highly inbred lines	−0.068	0.029	−2.38	0.018
Moderately inbred lines	−0.005	0.022	−0.21	0.83

Intercept equals outbred control lines; df (residual) = 423.

Neither sperm length (L-ratio for treatment = 2.74, df = 2, *P* = 0.25) nor sperm swimming speed (L-ratio for treatment = 0.12, df = 2, *P* = 0.94) differed among inbreeding treatments (intercept was the only term left in the final models of both variables), but these nonsignificant results are not due to low power. For both sperm length and swimming speed, we would have been able to detect a 10% change from the outbred treatments in both directions and a 5% change to the direction observed.

## Discussion

Our demonstration of strong inbreeding depression (87% for highly inbred lines and 65% for moderately inbred lines) in male attractiveness (i.e., mating latency) and mild inbreeding depression in male remating latency (6% for highly inbred lines) is consistent with earlier studies on *D. melanogaster* that have documented strong inbreeding depression in male mating ability (Brittnacher 1981; Sharp 1984; Partridge et al. 1985; Miller et al. 1993; Hughes 1995; Enders and Nunney 2010). In these studies, male mating ability was measured in competitive mating trials (N inbred males competing against N outbred males for N females and the proportion of offspring sired used as a measure of male mating ability). Our finding that mating latency is increased due to inbreeding may provide the explanatory mechanism for the low male mating ability observed in these earlier studies. This relationship appears to be widespread, as mating latency or male sexual motivation has similarly been shown to suffer inbreeding depression in several other species (Joron and Brakefield 2003; van Oosterhout et al. 2003; Mariette et al. 2006; Ala-Honkola et al. 2009; Ketola and Kotiaho 2010; Okada et al. 2011). Our findings are also consistent with previous studies that have inferred directional selection on male attractiveness (Hosken et al. 2008; Ilmonen et al. 2009; Bolund et al. 2010; Zajitschek and Brooks 2010; Okada et al. 2011; Pölkki et al. 2012).

Also in line with earlier studies (Hughes 1997; Konior et al. 2005; Zajitschek et al. 2009; Michalczyk et al. 2010; Simmons 2011), we found that inbreeding decreases the competitive fertilization success of males, which may indicate a history of directional selection for higher P_2_ in the LH_M_ population of *D. melanogaster*. Our results are further consistent with those of Zajitschek et al. (2009) and Robinson et al. (2009) in that they similarly found no decrease in male competitive fertilization success with moderate levels of inbreeding (theoretical *f* = 0.25) and a significant decline only associated with a higher level of inbreeding (theoretical *f* = 0.5 in this study and 0.59 in Zajitschek et al. 2009). Our P_2_ results were further consistent with an earlier study on the same base population (LH_M_) that found no response to directional selection on P_2_ (Bjork et al. 2007) indicating relatively little additive genetic variance for this trait due to past selection having fixed alleles that increase it. Furthermore, Hughes (1997) concluded that most of the genetic variation in this trait is dominance variance, which renders P_2_ a mostly nonheritable trait (e.g., Bjork et al. 2007), the variation of which is maintained predominantly by antagonistic pleiotropy and ejaculate × ejaculate and ejaculate × female interactions (Clark et al. 1999; Clark 2002; Bjork et al. 2007; Fiumera et al. 2007). Hughes (1997) and Fiumera et al. (2007) suggested P_2_ was under stabilizing selection, but our findings may suggest otherwise, at least historically, as traits under stabilizing selection should show little-to-no inbreeding depression (Lynch and Walsh 1998).

Of course, 3% inbreeding depression in P_2_ is not severe, but note that P_2_ in this study was lower in highly inbred males despite females taking longer prior to remating with such males compared to females remating with outbred control males (Table 1). This difference means that our test of the influence of inbreeding on P_2_ was particularly conservative, because females mated to highly inbred males should have used more of the first male's sperm prior to remating, a prediction that was supported by slightly greater numbers of offspring produced prior to remating by females that were remating with highly inbred compared to outbred control males (pair-wise difference, *P* = 0.053; Table 2). Hence, all else being equal, highly inbred males would be expected to have had higher P_2_ values than the outbred control males. Indeed, an earlier study using the same experimental material did find a positive correlation between the number of eggs produced prior to remating and P_2_ (see Table 2 in Ala-Honkola et al. 2011). We further consider the demonstrated inbreeding effect on P_2_ to be conservative as P_2_ values were unusually high experiment-wise (Fig. 2; 0.95 to 0.98, compared to previous reports of about 0.8: e.g., Morrow et al. 2005; Bjork et al. 2007; but see Clark et al. 1999). In our previous study on the same LH_M_-RFP population, P_2_ values were about 0.8 when competitor males were from a brown-eyed line (LH_M_*bw*^D^; Ala-Honkola et al. 2011). Hence, our standard competitor males were unusually uncompetitive, and it is likely that differences among inbreeding treatments would have been magnified had we used competitor males from a line better in sperm competition.

In contrast to male attractiveness and P_2_, we did not find inbreeding depression for sperm length, suggesting length has a history of stabilizing selection in this population. This result is surprising as sperm competition studies with *D. melanogaster* lines selected for increased or decreased sperm length (Miller and Pitnick 2002; Pattarini et al. 2006) or using isogenic lines derived from the same LH_M_ population as this study (Lüpold et al. 2012), have consistently demonstrated a long-sperm advantage, as manifested by a superior ability to displace, and to resist displacement by, competitor sperm. Although such a long-sperm advantage suggests that sperm length might be under directional selection, a hemiclonal analysis of the LH_M_ population concluded that sperm length is under stabilizing selection (Morrow et al. 2008). The lack of inbreeding depression in this study is consistent with that finding. The sperm length selection experiment (with a different population of *D. melanogaster*) showed that sperm length responds to intense bidirectional artificial selection (after 18 generations, increasing by 5.3% in the high line and decreasing by 7.9% in the low line), so there is substantial additive genetic variation for this character (a mean realized heritability of 0.48; Miller and Pitnick 2002). The lack of strong asymmetry in response to selection also suggests that this trait has not been under strong directional selection (Falconer and Mackay 1996).

True stabilizing selection tends to reduce genetic variation (Robertson 1956; Falconer and Mackay 1996), but given that empirical studies suggest that sperm length harbors ample genetic variance (e.g., Miller and Pitnick 2002; Morrow et al. 2008; Lüpold et al. 2012), it is likely that an intermediate optimum for sperm length arises from pleiotropic effects. For example, the production of longer sperm has been associated with substantive energetic and life-history costs (Pitnick and Markow 1994; Pitnick et al. 1995; Pitnick 1996), which could explain the net stabilizing selection (Falconer and Mackay 1996, pp. 344–347). The inbreeding method alone cannot distinguish weak selection from stabilizing selection as both scenarios predict little-to-no inbreeding depression for a trait (Lynch and Walsh 1998). However, we suggest that there is enough evidence for selection on sperm length from previous research (see above) to support our conclusion of the history of stabilizing selection on this trait in *D. melanogaster*.

In addition to sperm length, we simultaneously assayed numerous ejaculate characteristics with the dual goal of quantifying the extent of their inbreeding depression and of discerning the mechanisms underlying any treatment differences in competitive fertilization success. Although we were unable to confidently resolve the underlying causes of the reduced fertilization success in highly inbred males, we did demonstrate that it was not attributable to (1) fewer sperm per ejaculate, (2) a reduction in the number of sperm stored, or (3) lower sperm viability in the female reproductive tract but our statistical power to detect changes in sperm numbers was very low. Viability of inbred males’ sperm was measured indirectly from offspring production (measured over 10 days) by outbred females. Offspring production or offspring viability of females singly mated to inbred males did not differ from that of females mated to outbred males, suggesting that inbred males’ sperm survive equally well in the female reproductive tract and fertilize eggs as efficiently as sperm of outbred males. This contrasts with findings of inbreeding depression for male fertility in *D. simulans* (Okada et al. 2011), although Michalczyk et al. (2010) similarly found no difference in fertility or offspring viability of inbred (eight generations of full-sibling mating) flour beetle *Tribolium castaneum* males in a single-mating situation, despite inbred males having decreased competitive fertilization success.

When female *Drosophila* remate, some of the first male's sperm are released or displaced from the storage organs and eventually ejected by the female along with excess second-male sperm (Snook and Hosken 2004; Manier et al. 2010). Relatively slow and/or long sperm have been shown to be better at displacing resident sperm from storage (Lüpold et al. 2012), indicating selection for slower sperm. As no inbreeding depression was found in sperm swimming speed, either weak or stabilizing selection has acted on it (Lynch and Walsh 1998). We suggest that sperm swimming speed is likely to be under stabilizing selection. However, this requires confirmation.

Sperm competition success is a complex trait that may be affected by the number of sperm ejaculated (e.g., Boschetto et al. 2011), sperm mobility (e.g., Gage et al. 2004), sperm morphology (e.g., Oppliger et al. 2003; Lüpold et al. 2012), sperm viability (e.g., García-González and Simmons 2005), seminal fluid proteins (e.g., Chapman et al. 2000), female genotype (e.g., Wilson et al. 1997; Clark and Begun 1998; Birkhead et al. 2004), and ejaculate-female interactions (see review in Pitnick et al. 2009b). As we measured most of these parameters and found no differences between inbred and outbred males, we cannot presently explain the mechanism(s) underlying the reduced sperm competitiveness of highly inbred males. Unexamined mechanisms that could explain these findings include seminal fluid proteins and unknown aspects of female sperm choice (Eberhard 1996; Birkhead 1998; Pitnick and Brown 2000).

Directly assessing the history of selection on male ejaculate characteristics has proven difficult in most instances due to the large number of potential selective bouts that could define net selection, and because much of this occurs cryptically within the female reproductive tract. We have employed a novel method to assess selection and see this as a new way to provide insight into these key male fitness determinants. This study suggests that male attractiveness and possibly also competitive fertilization success (i.e., P_2_) have been under directional selection, whereas sperm length has a history of stabilizing selection.

## Supporting Information

**Figure S1.** Number of progeny (mean ± SE) males from different inbreeding levels sired during 10 days after a single mating with an outbred female; *N* = 39 in *f* = 0, *N* = 43 in *f* = 0.25, and *N* = 40 in *f* = 0.5.

**Figure S2.** Egg-to-adult viability (mean ± SE) of the progeny sired by males from different inbreeding levels (females were outbred), *N* = 40 in *f* = 0, *N* = 44 in *f* = 0.25, and *N* = 41 in *f* = 0.5.

**Table S1.** Final least squares model of offspring production of inbred males (first-order autoregressive variance covariance structure [AR1] and “day” as a variance covariate). df (residual) = 1212.

**Table S2.** Full least squares model of offspring production of inbred males (first-order autoregressive variance covariance structure (AR1) and “day” as a variance covariate). df (residual) = 1190.

**Table S3.** Full least squares model of offspring viability of inbred males (first-order autoregressive variance covariance structure [AR1]). df (residual) = 361.

**Table S4.** Full least squares model of sperm competition success (P_2_) of inbred males (first-order autoregressive variance covariance structure [AR1] and “treatment” as a variance covariate). df (residual) = 415.

Data deposited in the Dryad Repository doi: 10.5061/dryad.k7d00.
