# Supplementary material for: Inbreeding reveals mode of past selection on male reproductive characters in Drosophila melanogaster
Source: Ecol Evol. 2013 Jun 3;3(7):2089–102. doi: 10.1002/ece3.625 (PMC3728949; doi:10.1002/ece3.625)
Supplement: Supplementary file 1 [file ece30003-2089-SD1.docx]

## Supporting Information for the paper:

## “Inbreeding reveals mode of past selection on male reproductive characters in *Drosophila melanogaster”* by Outi Ala-Honkola et al.

#
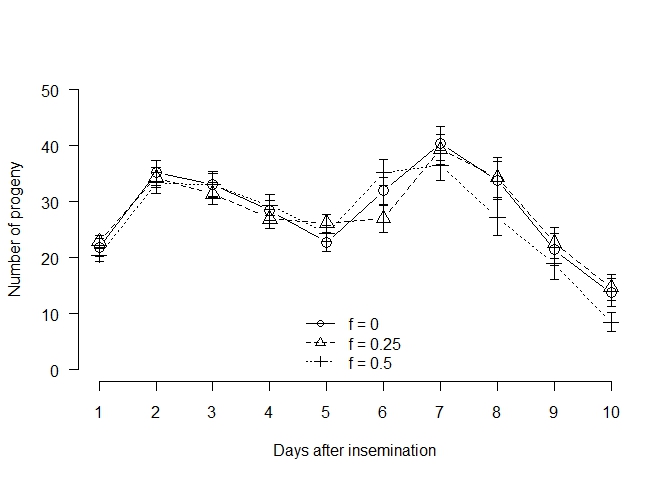


Figure A1. Number of progeny (mean ± SE) males from different inbreeding levels sired during 10 days after a single mating with an outbred female; N = 39 in f = 0, N = 43 in f = 0.25 and N = 40 in f = 0.5.


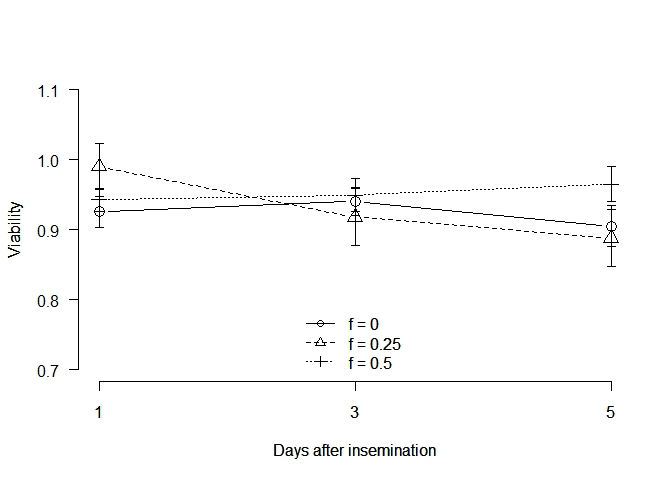


Figure A2. Egg-to-adult viability (mean ± SE) of the progeny sired by males from different inbreeding levels (females were outbred), N = 40 in f = 0, N = 44 in f = 0.25 and N = 41 in f = 0.5.

Table A1. Final least squares model of offspring production of inbred males (first order autoregressive variance covariance structure (AR1) and “day” as a variance covariate). Df(residual) = 1212.

| Effect | Parameter estimate | SE of the estimate | t | p |
| --- | --- | --- | --- | --- |
| Intercept | 27.7 | 0.62 | 44.9 | <0.000 |
| Day linear | -7.68 | 1.64 | -4.7 | <0.000 |
| Day ^2 | -15.0 | 1.32 | -11.0 | <0.000 |
| Day ^3 | -6.49 | 1.40 | -4.6 | <0.000 |
| Day ^4 | -9.30 | 1.24 | -7.5 | <0.000 |
| Day ^5 | 10.1 | 1.12 | 9.1 | <0.000 |
| Day ^6 | 3.55 | 1.08 | 3.3 | 0.001 |
| Day ^7 | -2.00 | 1.05 | -1.9 | 0.056 |
| Day ^8 | -3.03 | 0.95 | -3.2 | 0.001 |
| Day ^9 | 0.29 | 0.85 | 0.34 | 0.74 |

Table A2. Full least squares model of offspring production of inbred males (first order autoregressive variance covariance structure (AR1) and “day” as a variance covariate). Df(residual) = 1190.

| Effect | Parameter estimate | SE of the estimate | t | p |
| --- | --- | --- | --- | --- |
| Intercept | 6.99 | 13.7 | 0.51 | 0.61 |
| Male thorax | 0.022 | 0.13 | 0.17 | 0.86 |
| Female thorax | 0.258 | 0.16 | 1.6 | 0.11 |
| Highly inbred lines | -1.63 | 1.59 | -1.0 | 0.31 |
| Moderately inbred lines | -0.437 | 1.55 | -0.28 | 0.78 |
| Day linear | -6.23 | 2.92 | -2.1 | 0.033 |
| Day ^2 | -14.3 | 2.39 | -6.0 | <0.0001 |
| Day ^3 | -7.29 | 2.48 | -2.9 | 0.0034 |
| Day ^4 | -10.7 | 2.19 | -4.9 | <0.0001 |
| Day ^5 | 11.7 | 1.97 | 5.9 | <0.0001 |
| Day ^6 | 4.86 | 1.90 | 2.6 | 0.011 |
| Day ^7 | -2.24 | 1.84 | -1.2 | 0.22 |
| Day ^8 | -3.45 | 1.66 | -2.1 | 0.038 |
| Day ^9 | 1.39 | 1.47 | 0.95 | 0.34 |
| Day linear × Highly inbred lines | -5.06 | 4.11 | -1.23 | 0.22 |
| Day linear × Moderately inbred lines | 0.569 | 4.04 | 0.14 | 0.88 |
| Day ^2 × Highly inbred lines | -4.59 | 3.37 | -1.4 | 0.17 |
| Day ^2 × Moderately inbred lines | 2.35 | 3.31 | 0.71 | 0.48 |
| Day ^3 × Highly inbred lines | 2.48 | 3.49 | 0.71 | 0.48 |
| Day ^3 × Moderately inbred lines | -0.044 | 3.43 | -0.01 | 0.99 |
| Day ^4 × Highly inbred lines | 3.48 | 3.08 | 1.1 | 0.26 |
| Day ^4 × Moderately inbred lines | 0.57 | 3.02 | 0.19 | 0.85 |
| Day ^5 × Highly inbred lines | -2.08 | 2.77 | -0.75 | 0.45 |
| Day ^5 × Moderately inbred lines | -2.40 | 2.73 | -0.88 | 0.38 |
| Day ^6 × Highly inbred lines | -3.66 | 2.68 | -1.4 | 0.17 |
| Day ^6 × Moderately inbred lines | -0.315 | 2.63 | -0.12 | 0.90 |
| Day ^7 × Highly inbred lines | -2.69 | 2.58 | -1.0 | 0.30 |
| Day ^7 × Moderately inbred lines | 3.19 | 2.54 | 1.3 | 0.21 |
| Day ^8 × Highly inbred lines | 0.784 | 2.34 | 0.34 | 0.74 |
| Day ^8 × Moderately inbred lines | 0.481 | 2.30 | 0.21 | 0.83 |
| Day ^9 × Highly inbred lines | 1.70 | 2.06 | 0.82 | 0.41 |
| Day ^9 × Moderately inbred lines | -4.71 | 2.03 | -2.3 | 0.020 |

Table A3. Full least squares model of offspring viability of inbred males (first order autoregressive variance covariance structure (AR1). Df(residual) = 361.

| Effect | Parameter estimate | SE of the estimate | t | p |
| --- | --- | --- | --- | --- |
| Intercept | 1.12 | 0.31 | 3.7 | 0.0003 |
| Male thorax | -0.0016 | 0.003 | -0.56 | 0.57 |
| Female thorax | -0.0011 | 0.004 | -0.30 | 0.76 |
| Highly inbred lines | 0.033 | 0.030 | 1.1 | 0.27 |
| Moderately inbred lines | 0.034 | 0.029 | 1.2 | 0.24 |
| Day linear | -0.015 | 0.025 | -0.58 | 0.56 |
| Day ^2 | -0.020 | 0.020 | -1.0 | 0.31 |
| Day linear × Highly inbred lines | 0.031 | 0.035 | 0.87 | 0.38 |
| Day linear × Moderately inbred lines | -0.059 | 0.035 | -1.7 | 0.09 |
| Day ^2 × Highly inbred lines | 0.024 | 0.028 | 0.85 | 0.40 |
| Day ^2 × Moderately inbred lines | 0.038 | 0.028 | 1.4 | 0.17 |

Table A4. Full least squares model of sperm competition success (P_2_) of inbred males (first order autoregressive variance covariance structure (AR1) and “treatment” as a variance covariate). Df(residual) = 415.

| Effect | Parameter estimate | SE of the estimate | t | p |
| --- | --- | --- | --- | --- |
| Intercept | 1.13 | 0.33 | 3.4 | 0.0007 |
| Female thorax | 0.0044 | 0.0027 | 1.6 | 0.11 |
| 2^nd^ Male thorax | 0.0005 | 0.0035 | 0.13 | 0.90 |
| Day linear | 0.0011 | 0.015 | 0.07 | 0.94 |
| Day ^2 | -0.013 | 0.010 | -1.3 | 0.21 |
| Highly inbred lines | -0.085 | 0.027 | -3.1 | 0.0019 |
| Moderately inbred lines | -0.0074 | 0.027 | -0.27 | 0.79 |
| Day linear × Highly inbred lines | 0.0072 | 0.022 | 0.33 | 0.74 |
| Day ^2 × Highly inbred lines | 0.032 | 0.014 | 2.2 | 0.027 |
| Day linear × Moderately inbred lines | 0.014 | 0.022 | 0.62 | 0.53 |
| Day ^2 × Moderately inbred lines | 0.015 | 0.015 | 1.0 | 0.31 |
